# What Predicts Early Math in Autism? A Study of Cognitive and Linguistic Factors

**DOI:** 10.1007/s10803-025-06726-x

**Published:** 2025-01-31

**Authors:** Raúl Fernández-Cobos, Irene Polo-Blanco, Elena Castroviejo, Maria Juncal-Ruiz, Agustín Vicente

**Affiliations:** 1 Department of Mathematics, Statistics and Computer Science, Universidad de Cantabria, Avda. de los Castros 48, Santander, 39005 Spain; 2 Department of Linguistics and Basque Studies, Universidad del País Vasco (UPV/EHU), Paseo de la Universidad 5, Vitoria-Gasteiz, 01006 Spain; 3 Department of Psychiatry (Child and Adolescent Mental Health Unit), Sierrallana Hospital, Torrelavega, Spain; 4 Department of Medicine and Psychiatry, Instituto de Investigación Marqués de Valdecilla (IDIVAL), Universidad de Cantabria, Avda. Cardenal Herrera Oria s/n, Santander, 39011 Spain; 5 Ikerbasque – Basque Foundation for Science, Bilbao, Spain

**Keywords:** Autism, Language, Mathematics, Non-verbal intelligence

## Abstract

This study aimed to examine early mathematical abilities in young children with autism aged four to seven without intellectual disabilities and their connection with autism severity, non-verbal intelligence, and linguistic abilities (receptive vocabulary and grammar). The study involved 42 children with autism. We assessed participants’ cognitive, mathematical, and linguistic abilities. Their mathematical performance was compared with that of typically developing children using standardized measures. Statistical analyses were conducted to identify potential cognitive or linguistic differences across groups based on mathematical performance, and to determine predictive factors for mathematical abilities in children with autism. The findings indicated a higher prevalence of mathematical difficulties among the participants compared to typically developing children. A classification based on mathematical performance revealed statistically significant differences in cognitive and linguistic variables across groups, particularly in the low-performance group. However, no significant differences were found according to autism severity between the groups. The analysis further identified that a combination of visuo-spatial and linguistic abilities was the most predictive factor for mathematical performance. The study suggests that young children with autism without intellectual disabilities may be more likely to experience mathematical difficulties compared to typically developing children. Assessing cognitive and linguistic abilities could serve as a predictive measure for mathematical difficulties of children with autism, even without a formal diagnosis. Future research, with larger samples or longitudinal approaches, could validate these findings or explore which specific mathematical abilities are more related to non-verbal intelligence and which ones to structural language.

Individuals with autism, contrary to common belief, usually face challenges rather than strengths in general mathematical skills (Dowker, 2020). It has been reported that approximately one-fourth of students diagnosed with autism spectrum disorder (ASD) without intellectual disabilities (ID) are expected to experience difficulties with mathematical learning (Mayes & Calhoun, 2006). These difficulties are often attributed to various factors, including language comprehension, phonological awareness, verbal and spatial working memory, long-term memory, and executive functions (Dowker, 2020). While previous research has focused on assessing mathematical abilities in the population with autism (e.g., Kljajevic, 2023; Oswald et al., 2016; Polo-Blanco et al., 2024; Tonizzi & Usai, 2024), there is relatively limited research on early mathematical abilities in children with autism. On the other hand, the connection between non-verbal intelligence, language, and arithmetic has been extensively explored both theoretically (Carey, 2009; Dehaene, 2011; Spelke, 2017) and experimentally (Alt et al., 2014; Bialystok & Codd, 1997; Negen & Sarnecka, 2012; Praet et al., 2013) in typical development, but not in atypical profiles such as autism.

## Early Mathematical Abilities

Early mathematical abilities serve as the foundation for broader mathematical skills. Some early mathematical skills that have attracted the attention of researchers are: number estimation, the cardinality principle (counting), subitizing, quantity comparison, and basic addition and subtraction (Ginsburg & Baroody, 2003). Number estimation involves discerning quantities following Webber’s law: small differences in small quantities are easily discriminated, whereas discriminating differences in larger quantities requires larger differences. Understanding the cardinality principle, or counting, entails recognizing that the final number in a counting sequence represents the quantity of objects in a set. Subitizing refers to the ability to instantly recognize the number of objects in a display without counting. Quantity comparison involves determining which display has more or fewer objects, as well as identifying which number is larger or smaller, requiring mastering the comparative construction in the corresponding languages. Simple addition and subtraction imply combining or removing small quantities; such as determining the total after adding or subtracting one, two or three objects.

### Early Mathematical Abilities in Children with Autism

Research on the mathematical abilities of individuals with autism yields varied outcomes, depending on participants’ age (Aagten-Murphy et al., 2015; Fernández-Cobos & Polo-Blanco, 2024; Miller et al., 2017; Titeca et al., 2014, 2015, 2017; Wei et al., 2015). Titeca et al. (2014) observed 33 children with autism without ID from preschool to first grade, comparing them with 54 typically developing (TD) children. They found that during preschool years, early numeracy skills of the children with autism resembled those of TD peers, with verbal subitizing and counting predicting subsequent mathematical proficiency. Specifically, verbal subitizing was suggested to have a higher predictive value in children with autism compared to TD children. In a more recent article, Titeca et al. (2017) compared the early mathematical abilities of 20 children with autism (aged four and five) without ID to 20 TD controls. No significant group differences were observed across five early mathematical abilities (verbal subitizing, counting, magnitude comparison, estimation, and arithmetic operations), suggesting that later mathematical performance differences between children with and without autism may not stem from variations in foundational mathematical abilities. Similar results were reported by Titeca et al. (2015) when comparing 30 preschoolers with autism without ID and 30 age-matched children without autism in terms of the same abilities. Moreover, the authors observed no significant correlations between autism severity (measured by a social responsiveness scale) and early numerical competencies.

In contrast to Titeca and colleagues, Aagten-Murphy et al. (2015) found significant differences between children with and without autism (ages 8 to 13) in symbolic and non-symbolic magnitude estimation, with individuals with autism performing worse. Non-symbolic estimation is what we have referred above as “number estimation,” and symbolic estimation involves locating numbers in a spatial representation, such as a number line. While symbolic estimation correlated with mathematical performance in both groups, non-symbolic estimation did not. However, note that in other studies with TD children (Anobile et al., 2013) non-symbolic estimation appears as a predictor of subsequent mathematical abilities.

In a longitudinal study, Miller et al. (2017) assessed 26 children with autism around ages 2, 4, and 10 to investigate whether cognitive abilities, adaptive skills, and severity of ASD symptoms observed during preschool and school years accounted for a significant amount of academic achievement variance in later years. Results showed weakness in mathematics skills among children with autism, with early motor functioning predicting later mathematical abilities. Individuals with more severe ASD symptoms tended to exhibit lower academic abilities, particularly in reading, numerical operations (including counting and number identification), and math reasoning (involving problem-solving). However, concurrent intelligence quotients (IQ) significantly influenced academic achievement beyond adaptive skills and severity of ASD symptoms.

More recently, Li et al. (2023) assessed magnitude representation precision using an approximate number comparison task (dot comparison) in 70 preschool children with autism and 117 TD children. Children with autism showed lower numerical comparison accuracy, indicating weaker magnitude representation, compared to their TD peers. This difference in numerical comparison accuracy persisted even after accounting for various general cognitive abilities (working memory, inhibitory control, and non-verbal intelligence) and language abilities. In a similar vein, Wei et al. (2015) examined 130 children with ASD aged 6–9 and categorized them into four profiles based on reading, mathematical, cognitive, and social skills. Only 38.5% of the students demonstrated average achievement in mathematics (applied problems and calculation), while the remaining ones scored below expectations. In a study by Chen et al. (2019) involving 114 male children with ASD without ID aged 7–12, two academic achievement profiles were identified. While average achievement scores were within the normal range, one group (36.8% of the sample) exhibited poorer mathematical skills compared to reading skills, while the other group (63.2% of the sample) showed superior math skills compared to reading skills. In the same line, Fernández-Cobos & Polo-Blanco (2024) compared the mathematical abilities of 17 children with autism without ID in first to fourth grades and those of 17 TD students (matched by age, grade, and classroom) using the Test of Early Mathematical Abilities (TEMA-3; Ginsburg & Baroody, 2003). Participants with autism encountered more difficulties in mathematics, both in formal and informal aspects, compared to their peers. These differences became more pronounced with age, though were present in earlier grades for informal skills.

### Early Arithmetic Abilities, Language, and Non-verbal Intelligence

Developmental psychologists (Carey, 2009; Spelke, 2017) have argued for a role of language in arithmetic, particularly in the acquisition of the cardinality principle. In Carey’s case, mastering quantifiers (*one*, *some*) and the singular/plural morphology of language helps children infer that numbers name quantities (Carey, 2009; Negen & Sarnecka, 2012; Ansari et al., 2003). According to others (Condry & Spelke, 2008; Spelke, 2017), the involvement of language runs somewhat deeper: children learn the cardinality principle through understanding *phrases* containing numerals *one*, *two*, and *three*, and quantifiers, which provide them the means to build a unitary system of natural number concepts from a set of conceptual primitives delivered by core cognitive systems (the analogical number system and the tracking system that allows humans and animals to track up to four objects). In Spelke’s view, number concepts are intimately linked to number words, and how some of these number words behave in grammar.

The evidence for some involvement of language in inferring the cardinality principle is quite robust (see Praet et al., 2013; Spelke, 2017), although several studies minimize the role of language, finding relations only between vocabulary breadth (and not language as such) and early mathematics (e.g., Ansari et al., 2003; Purpura et al., 2011; Purpura & Ganley, 2014, on William Syndrome children). Actually, some researchers suggest that the main (and probably sole) role of language concerning early mathematics is to provide a symbolic system, regardless of its combinatorial properties, highlighting the verbal nature of number words but unrelating it to other linguistic elements or features (e.g., Kolkman et al., 2013). Once children have acquired a symbolic system and understood how symbols work, they can begin to hypothesize what the number words are for. On the other hand, it is important to note that some studies have found little support for the involvement of language in early mathematical abilities, including mastering the cardinality principle. Thus, Yang and Liang (2022) delved into the understanding of cardinal number words in Mandarin Chinese-speaking children aged two to five. The study also assessed their general language proficiency, intelligence, approximate number system acuity, and knowledge of quantifiers. The findings indicated that domain-specific numerical skills appeared to play a more crucial role in children’s development of cardinal number words compared to the more immediate domain-general abilities such as language skills and intelligence.

However, not all early mathematical abilities are supposed to relate to language (be it grammar or vocabulary) in the same way. The focus of studies concerning language and mathematics has been the cardinality principle, but language is not supposed to predict approximate number estimation (Spelke, 2017). Thus, LeFevre et al. (2010) tested a model to examine the relationships among early numeracy skills, mathematical outcomes, and three distinct cognitive precursors: quantitative (including non-numerical and numerical comparison and subitizing), linguistic, and spatial attention, for 182 children aged four to seven years. Linguistic abilities were assessed using receptive vocabulary and phonological awareness measured by the Peabody Picture Vocabulary Test (PPVT-III; Dunn & Dunn, 1997) and Comprehensive Test of Phonological Processing (Wagner et al., 1999), respectively. Early numeracy measures included number naming and nonlinguistic arithmetic measures, the latter involving the representation and mental manipulation of quantities without labeling them with symbols. The study revealed that linguistic skills uniquely predicted variability in number naming but not in nonlinguistic arithmetic. Spatial attention emerged as a unique predictor of variability in both early numeracy measures. Similarly, Zhang (2016) investigated the connection between language, visual-spatial skills, executive functions, and the early grasp of numerical concepts in 109 Chinese 3-year-old children. The researchers found that vocabulary, letter knowledge, spatial perception, and executive skills each played a distinct role in number competence.

The possible involvement of language in early mathematical skills has also been investigated by testing children with linguistic difficulties (Alt et al., 2014; Ansari et al., 2003; Desoete & Warreyn, 2020). For example, Alt et al. (2014) explored the relation between mathematics and language in children (aged 6–9) with developmental language disorder (DLD), compared to English language learners. While mathematical difficulties observed in English language learners seemed to stem from the language requirements of mathematical tasks, children with DLD struggled with mathematical tasks due to both linguistic and non-linguistic processing constraints. In the same line, Koponen et al. (2006) investigated numerical abilities in 29 children with DLD and 20 TD children from preschool to third grade and the extent to which linguistic factors accounted for variations in these skills. Children with DLD lagged behind their educational age counterparts in both verbal and non-verbal numerical skills. A connection between the ability to recall arithmetic facts and naming fluency was found, while differences in non-verbal numerical skills were not explained by measured cognitive skills (non-verbal reasoning skill, verbal short-term memory, vocabulary, comprehension, and naming fluency).

Interestingly for our purposes, Ansari et al. (2003) investigated the relationship of the cardinality principle with language and visuo-spatial cognition in children with Williams Syndrome (mean age seven years old) and TD children (mean age three and a half) respectively. In the Williams Syndrome group, language was the sole factor explaining a significant portion of the variance in cardinality understanding. In contrast, in the TD comparison group, only visuo-spatial ability predicted the variance. This study suggests that language may have a differential impact depending on the condition that children exhibit.

### Research Questions

Previous research on TD children has examined the link between early mathematical skills and language, with some studies focusing on vocabulary (Yang & Liang, 2022; Zhang, 2016) and others on other language skills (Praet et al., 2013; Purpura et al., 2011; Toll & Van Luit, 2014; Viesel-Nordmeyer et al., 2022). In the case of studies involving children with autism, there is a scarcity of research evaluating early mathematical learning. Most studies have focused solely on exploring connections between different mathematical skills (Aagten-Murphy et al., 2015; Fernández-Cobos & Polo-Blanco, 2024; Titeca et al., 2014, 2015, 2017); some have examined links between mathematical and cognitive or academic skills without emphasizing language (Chiang & Lin, 2007; Miller et al., 2017), and very few have taken language into consideration (Li et al., 2023).

The purpose of our study is to test early mathematical abilities in autism in relation to different clinical, cognitive and linguistic variables^1^. We hypothesized that children with autism would perform worse than TD children in early mathematical tasks. Although literature results vary, developmental delays likely contribute to lower mathematical abilities, even with typical non-verbal intelligence. Based on other researchers’ findings, we also expected connections between linguistic variables and mathematical performance, although it is uncertain whether vocabulary or grammar would have stronger associations due to limited research. Non-verbal intelligence was hypothesized as a predictor of mathematical performance, with evidence suggesting a link between visuo-spatial scores and early mathematical performance in TD children (Ansari et al., 2003; Holmes et al., 2008) Lastly, autism severity was hypothesized as a predictor of mathematical difficulties; while Titeca et al. (2017) found no correlation, Miller et al. (2017) did, suggesting a potential connection between symptom severity and mathematical abilities due to its impact on developmental progress. Specifically, we address the following questions:

1. Does the distribution of our sample differ in terms of mathematical performance in relation to what is expected in TD children?
2. Are there statistically significant differences in the clinical, cognitive or linguistic variables between groups of mathematical performance?
3. Which clinical, cognitive or linguistic variables predict the mathematical performance in our sample?

## Methods

### Participants

The study involved 42 children with autism (37 males and 5 females; average age 6.17, with standard deviation 1.00 and range of values [4.2–7.8]), recruited between January and June 2023 from various sources dedicated to supporting individuals with autism in the Spanish regions of Álava and Cantabria, including child psychiatry and pediatric outpatient clinics, family associations, and school counseling professionals. The inclusion criteria were: (1) ASD diagnosis without other psychiatric comorbidities, as per the Diagnostic and Statistical Manual of Mental Disorders (DSM-5; American Psychiatric Association, 2013); (2) non-verbal IQ (NVIQ) ≥ 70 as measured by the Leiter-3 non-verbal intelligence scale (Koch et al., 2019; Roid & Miller, 2013); (3) aged 4–8; and (4) comprehension of brief instructions and the ability to point.

Initially, we selected 51 children previously diagnosed with ASD at mental health units. A child psychiatrist reviewed their medical records, confirming ASD diagnoses per DSM-5 guidelines and absence of comorbidities, based on parent interviews and patient evaluations. Nine children did not meet the inclusion criteria: two had comorbidities with attention deficit/hyperactivity disorder (ADHD), seven were excluded for not demonstrating comprehension of brief instructions or not displaying the ability to point.

After explaining the study, parents or legal guardians consented by signing forms. This research had approval from the Ethics Committee for Clinical Research of Cantabria (CEIC-C) and the University of the Basque Country’s Ethics Committee for Research Involving Human Beings (CEISH).

### Measurement Variables and Instruments​​

Autism severity was assessed using the chart from Gotham et al. (2009, p. 699 [Table 2]), which gathers ADOS-2 (Lord et al., 2012) modules 1, 2, and 3 for ages 2–16. Scores are mapped from ADOS-2 modules, age, and score received in the ADOS-2 to a severity score, ranging from 1 to 10: 1–3 as NS (“Non-spectrum”), 4–5 as ASD (“Autism Spectrum Disorder”), and 6–10 as AUT (“Autistic”).

Non-verbal intelligence was measured with the Leiter-3 scale (Koch et al., 2019; Roid & Miller, 2013), which must be fully administered without verbal instruction or requiring verbal responses from participants. It comprises three sets of subtests: Fluid Intelligence subtests, Attention and Memory subtests, and Social/Emotional scale. The subtests comprising Fluid Intelligence provide a non-verbal intelligence quotient (NVIQ). Within Fluid Intelligence, two groups of subtests can be formed: Visuo-spatial abilities (Figure Ground, and Form Completion), and Reasoning (Classification and Analogies, and Sequential Order). Figure Ground consists in finding figures within visual displays of increasing complexity, while Form Completion requires the participant to mentally ensemble parts of a geometrical display. Concerning Reasoning, Classification and Analogies taps onto reasoning by analogy, while Sequential Order is a task similar to Raven’s matrices, measuring the meaning making ability of participants. The children’s NVIQ distribution within our sample was typical, with five participants (11.9%) falling within the 70–85 range, compared to an expected 13.5% in a normal distribution with zero mean and a standard deviation of 15 (Roid & Miller, 2013: p. 159). Symmetrically, other five participants had a NVIQ between 115 and 130.

Receptive vocabulary skills were assessed using the PPVT-III (Spanish version by Dunn et al., 2010). This test can be administered to children of ages starting at two years and six months. This is a pointing task, whereby the experimenter utters a target vocabulary item, and the child has to point at the right pictures given four alternatives. There are 16 blocks of 12 items each, ordered by difficulty. Correct and incorrect answers are collected until eight mistakes are made within the same block, which leads to the end of the test. The test is calibrated according to the breadth of vocabulary at each Chronological Age (CA) in typical populations, providing both a direct score and an estimated verbal mental age (VMA). Receptive vocabulary was transformed in this study as (VMA-CA)/CA for test comparison.

Grammatical skills were evaluated using the Spanish Test of Comprehension of Grammatical Structures (CEG; Mendoza et al., 2005a), a grammatical scale inspired by Bishop’s (2003) Test for Reception of Grammar (TROG-2; see, for the English language, Mendoza et al., 2005b). CEG is a clinical tool for children from 4 to 11 years old, and consists of 20 blocks, each one identifying one linguistic structure of Spanish as defined by the authors of the test. It is thus not strictly speaking a tool of evaluation of grammatical competence, but rather of command of specific grammatical structures. Each block is composed of four items exemplifying each structure. These include transitive constructions (reversible and non-reversible depending on whether it is sensible to analyze the first constituent as the subject or object), copular sentences, sentential negation, clefts, clitic left dislocation structures, or relative clauses of different sorts, to name a few examples. In this test, the experimenter reads a target sentence and the child has to point at the right picture in view of four alternatives, much like in the PPVT-III. All children complete the entire test and the number of errors is counted. In relation to this, unlike what happens with the TROG-2, here blocks are not ordered by degree of complexity, even if the first sets of blocks illustrate simpler syntactic structures than the last ones. For our analysis, we collected the number of correct blocks by each child and computed *z*-scores based on expected values and standard deviations for different age intervals given by Mendoza et al. (2005b).

Finally, early mathematical abilities were evaluated using the TEMA-3 (Ginsburg et al., 2007; Ginsburg & Baroody, 2003), designed for children aged 3 to 9. The instrument’s internal consistency has been reported at 0.90 for TD population (Ginsburg & Baroody, 2003) and the test has been employed in studies involving children with intellectual and developmental disabilities (e.g., Vostanis et al., 2021) and autism (Fernández-Cobos & Polo-Blanco, 2024; Polo-Blanco et al., 2024). This performance-based test comprises 72 items that assess both formal (31 items) and informal (41 items) mathematical skills. Within informal skills, the following categories are distinguished: (1) numbering (subitizing, mastery of numerical sequence through tasks involving basic counting and enumeration skills, cardinality principle), (2) comparison (ability to establish relative distances between numbers, order of the numerical sequence), (3) calculation (strategies supported by counting, with and without concrete objects, and non-verbal mental calculation skills), and (4) concepts (numerical constancy, application of advanced counting strategies, basic understanding of object distribution, part-whole relationship). As it can be seen, TEMA-3 evaluates several relevant mathematical skills, although such skills do not need to constitute a completely exhaustive inventory of the early mathematical skills. Test direct scores (*DS*) range from 0 to 72, with one point awarded for each correct item, ending after five consecutive incorrect responses. To compare mathematical performance with that expected in TD children, we computed *z*-scores as $\:\left(DS-\stackrel{-}{DS}\right)/\sigma\:\left(DS\right)$, using expected values ($\:\stackrel{-}{DS}$) and standard deviations ($\:\sigma\:\left(DS\right)$) for each age interval from the Spanish standardization sample in Ginsburg et al. (2007, p. 88). Although TEMA-3 provides a mathematical competence index (MCI), which is a standardized variable, we decided to use *z*-scores because they are more sensitive at low and high performance (MCI saturates at 55 and 150, corresponding to $\:\pm\:3\sigma\:$). Since the statistical information is not available for separate scores of informal and formal skills, relative differences between the obtained score and the expected score for the participant’s age were considered for informal and formal total scores, and for numbering (which includes a significant proportion of the informal items). The small number of items for the rest of categories at small ages makes no possible a robust analysis.

### Analysis

We classified mathematical performance into three levels (low, medium and high) based on TEMA-3 *z*-scores using $\:\pm\:1.5\sigma\:\:$thresholds. The *z*-scores allows us to compare the distribution of mathematical competence in our sample with that expected from TD children without requiring a control group, for instance, examining whether the number of children within the low or high-performance groups significantly differs from the TD expectations. To address the second research question, we analyzed clinical, cognitive and linguistic variables across groups of mathematical performance (hereinafter, LMP, MMP and HMP, for low, medium and high mathematical performance). For instruments yielding direct scores expected to increase with age in TD children (TEMA-3, PPTV-III, and CEG) we employed standardized variables (*z*-scores or relative differences). ANOVA tests were employed to compare the average of each variable across different mathematical performance groups, with Bonferroni correction applied to minimize type-I error in multiple comparisons. Statistically significant differences between mathematical performance groups would suggest that mathematical performance may explain the distribution of the corresponding variable in our sample.

On the other hand, Pearson’s correlation test with Bonferroni correction was used to examine which clinical, cognitive and linguistic variables may be related with mathematical performance, and also to investigate whether the possible associations are more linked to informal or formal mathematical skills. Guided by this previous exploration, a predictive model was built using Stepwise Regression (Harrell, 2015) to identify variables with the strongest predictive power for mathematical performance. Variables are sequentially added in the model based on highest correlation with the dependent variable (mathematical performance). An independent variable is entered into the equation only if it meets the entry criterion (in this case, *p* < 0.05). Variables already entered in the regression equation can be removed from the model if they meet the output criterion (*p* > 0.10). The method terminates when there are no more candidate variables to include or eliminate.

## Results

Table 1 summarizes the characteristics of our sample, considering clinical, cognitive, linguistic and mathematical variables.

**Table 1:** Comparisons between groups of mathematical performance

	Cognitive			Language	
	NVIQ	Visuo-spatial abilities	Reasoning	PPVT	CEG
MMP vs. LMP	0.002**	0.003**	0.004**	0.003**	0.004**
HMP vs. LMP	< 0.001**	0.001**	< 0.001**	0.003**	0.011*
HMP vs. MMP	0.043*	0.286	0.103	0.560	1.000

Note. *<0.05. **<0.01

**Fig. 1:**
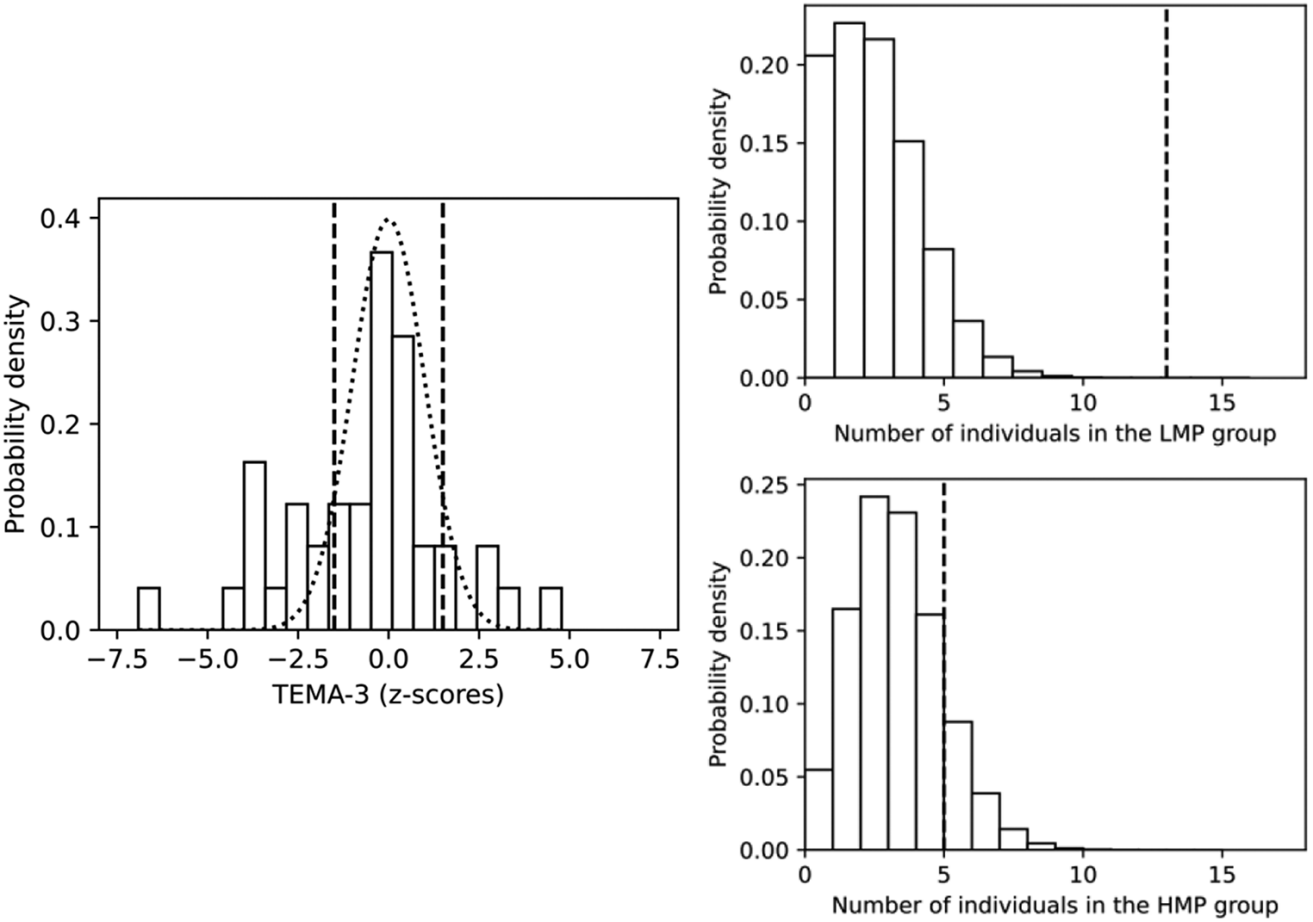
Distribution of our sample according to mathematical performance

Starting with the distribution of mathematical performance, the left panel in Fig. 1 shows the distribution of *z*-scores for TEMA-3 in our sample. 13 of 42 children are in the LMP group, five are included in the HMP group, and the remaining 24 belong to the MMP group. The dotted curve represents the expected distribution for TD children, according to which, on average, 6,68% of the children would be expected to present LMP for their age, and another 6,68% to present HMP. Right panels in Fig. 1 show the distribution of the number of children within the LMP (upper panel) and HMP (bottom panel) groups for 10^8^ random samples of 42 TD children. Dashed vertical lines indicate the number of children found within our sample of children with autism. While there would be a probability of 0.147 of getting 5 or more individuals in the group of HMP in a random sample of 42 TD children, there would be only a probability of 2 × 10^− 6^ of getting 13 or more individuals in the group of LMP.

**Fig. 2:**
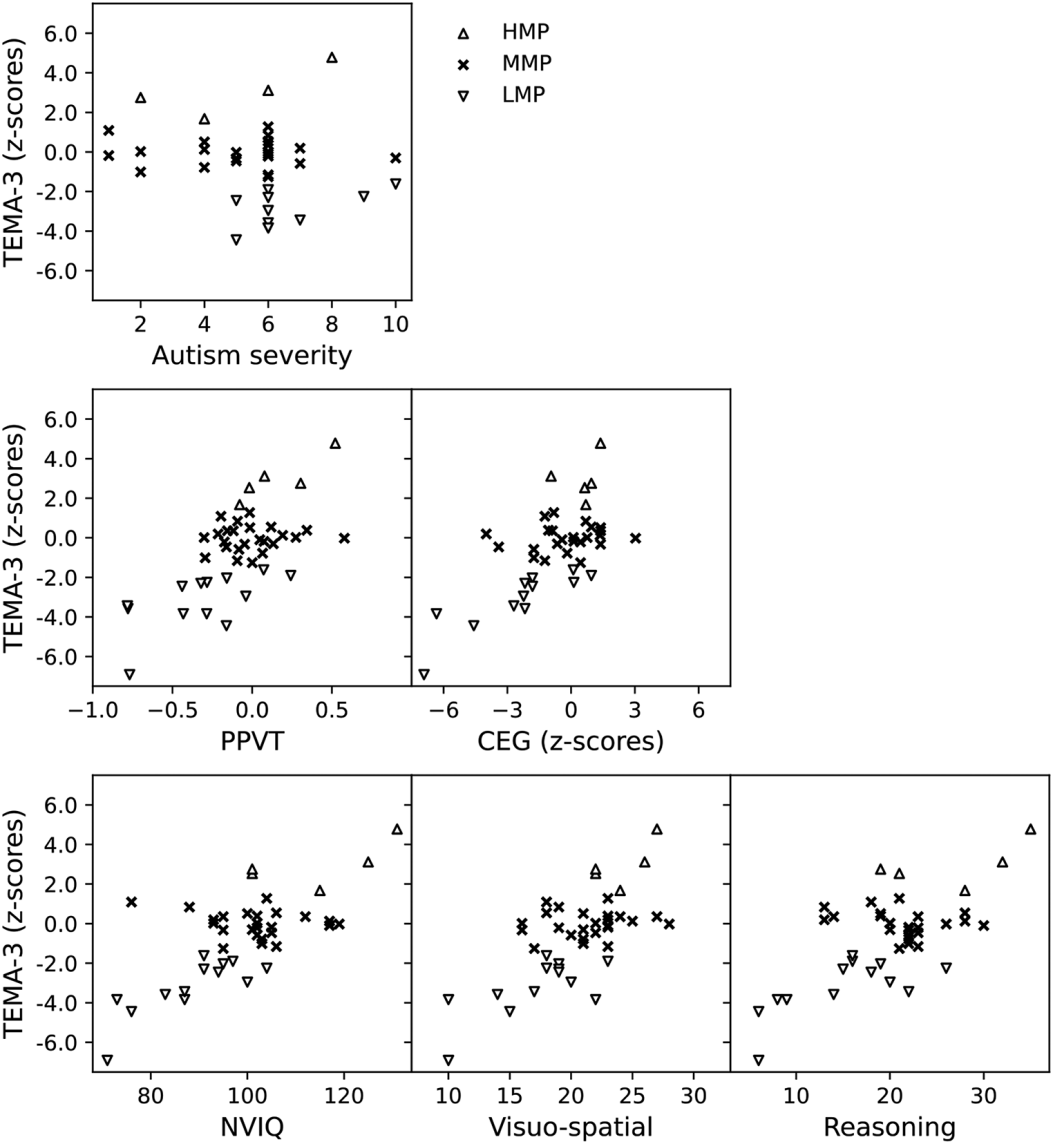
Distribution of clinical, linguistic and cognitive variables according to groups of mathematical performance

Figure 2 offers a visual representation for observing the distribution of each clinical, cognitive and linguistic variable in relation to the groups of mathematical performance. For those cases in which the difference in mean values between groups of mathematical performance showed to be statistically significant, Table 2 shows the *p*-values obtained from a post-hoc multiple comparison analysis. This revealed statistically significant differences between the mean values of all the cognitive and linguistic variables considered of individuals within MMP and LMP groups, and between HMP and LMP groups. Differences between HMP and MMP groups are statistically significant only for NVIQ. No statistically significant differences of autism severity were found between groups of mathematical performance.

**Table 2:** Clinical, cognitive, linguistic, and mathematical data

	Mean	Standard deviation	Range of values
Clinical data			
Autism severity	5.50	2.06	1–10
Cognitive data			
NVIQ	99.12	13.02	71–131
Visuo-spatial abilities	20.43	4.03	10–28
Reasoning	20.05	6.42	6–35
Linguistic data			
PPTV-III relative difference of VMA	-0.08	0.30	-0.78–0.58
CEG *z*-scores (number of correct blocks)	-0.78	2.10	-6.92–3.03
Mathematical abilities			
TEMA-3 *z*-scores	-0.65	2.20	-6.91–4.78
Mathematical age	5.98	1.25	3.8–9.0
Relative difference of total informal	-0.01	0.44	-0.86–1.54
Relative difference of informal numbering	0.02	0.42	-0.77–1.33
Relative difference of total formal	0.37	1.45	-1.00–8.00

Note. Data were collected from 42 participants, with autism severity data for 38. NVIQ denotes non-verbal intelligence quotient. Linguistic variables include relative differences between estimated verbal mental age (VMA) from PPVT-III and chronological age, and z-scores for correct blocks from CEG. Mathematical abilities include z-scores for total direct scores from TEMA-3, and relative differences for total informal, numbering, and formal direct scores

Figure 2 allows also a visual inspection of correlations involving *z*-scores from TEMA-3. As shown in Table 3, all correlations involving *z*-scores were moderate with high statistical significance after Bonferroni’s correction (*p* < 0.001), except for autism severity. Correlations involving relative differences of informal numbering and informal and formal total scores are also shown.

**Table 3:** Correlation coefficients ($\:\rho\:$) between clinical, cognitive and linguistic variables and TEMA-3

	Total		Informal		Informal numbering		Formal	
	$\:{\uprho\:}$	*p*-value	$\:{\uprho\:}$	*p*-value	$\:{\uprho\:}$	*p*-value	$\:{\uprho\:}$	*p*-value
Autism severity	-0.17	1.000	-0.09	1.000	-0.13	1.000	0.04	1.000
NVIQ	0.70	< 0.001**	0.51	0.025*	0.67	< 0.001**	0.60	0.001**
Visuo-spatial abilities	0.68	< 0.001**	0.38	0.614	0.58	0.003**	0.50	0.034*
Reasoning	0.67	< 0.001**	0.50	0.036*	0.69	< 0.001**	0.56	0.005**
PPVT	0.67	< 0.001**	0.46	0.106	0.57	0.003**	0.54	0.012*
CEG	0.67	< 0.001**	0.33	1.000	0.55	0.008**	0.42	0.313

Note. * <0.05. ** <0.01. Pearson’s correlation test after applying Bonferroni’s correction

Based on the obtained results, the Stepwise Regression considered NVIQ, PPTV-III and CEG. It yielded a model (Model 1 in Table 4) preserving only NVIQ and CEG as predictors, explaining 54% of the variability in the mathematical performance *z*-scores. Considering that NVIQ could be an important predictor, we built a finer-grained version of the model to determine if any of the two components (visuo-spatial abilities and reasoning) plays a more important predictive role than the other. In this case, the Stepwise Regression considered NVIQ, visuo-spatial abilities, reasoning, PPTV-III and CEG. The obtained model (Model 2 in Table 4) preserved only visuo-spatial abilities and CEG as predictors, explaining 58% of the variability in mathematical performance.

**Table 4:** Predictive models from the stepwise regression for Z-scores from TEMA-3

Model	Predictors	Coefficients			Model summary		
		β	t	*p*	F	*p*	Adjusted R^2^
1	NINV	1.08	3.21	0.003	24.30	< 0.001**	0.538
	CEG	1.48	2.71	0.010			
2	Visuo-spatial abilities	1.30	3.90	< 0.001	28.65	< 0.001**	0.580
	CEG	1.46	2.89	0.006			

Note. * <0.05. ** <0.01

## Discussion

In this study, we have examined cognitive, linguistic, and early mathematical skills of 42 children with autism. Our first research question addressed the distribution of mathematical performance within this sample. Our findings revealed lower performance among participants with autism compared to age expectations in typical development (Ginsburg et al., 2007; Ginsburg & Baroody, 2003), consistent with prior studies that found lower mathematical performance in children with autism relative to their TD peers (e.g., Aagten-Murphy et al., 2015; Fernández-Cobos & Polo-Blanco, 2024; Lie et al., 2023; Wei et al., 2015). These results contrast with studies that reported no significant differences in mathematical abilities between groups with and without autism (Titeca et al., 2014, 2015, 2017). However, while overall mathematical performance among children with autism tended to be below age expectations, a subset performed at or above age level, consistent with previous findings indicating diverse profiles of mathematical performance (e.g., Chen et al., 2019). This suggests that differences between our results and those of Titeca and colleagues may relate to the nature of the sample, which, at least in the case of the current study, was quite heterogeneous.

ANOVA tests were conducted to address the second research question, focusing on clinical, cognitive or linguistic variables that could be affected by the distribution of mathematical performance in our sample. First, the mean values for autism severity did not differ significantly depending on the group of mathematical performance of participants, which contrasts with the potential relation between autism severity and academic achievement reported by Miller et al. (2017). Secondly, linguistic variables showed to be statistically different between the low mathematical performance (LMP) group and the other two groups of medium and high mathematical performance (MMP and HMP). Thirdly, although significant differences were observed in mean non-verbal intelligence quotient (NVIQ) among all groups of mathematical performance, we found visuo-spatial abilities and reasoning significantly different only between the LMP and the rest of groups of mathematical performance, as occurs with the linguistic variables.

In relation to the third research question concerning predictors of early mathematical abilities, our results suggested that the strongest predictor of success in early mathematical abilities in our sample is a combination of visuo-spatial and linguistic (grammar) abilities, which are variables differentiating the LMP group. This could mean that, in this respect, the population with autism is similar to the population without autism, given the evidence that visuo-spatial and linguistic skills are good predictors of early mathematical skills in TD children (e.g. LeFevre et al., 2010; Zhang, 2016). As early mathematical abilities include different skills (approximate number estimation, counting, subitizing, quantity comparison, and simple addition and subtraction), our results suggest that some skills may be better predicted by visuo-spatial abilities and other by linguistic abilities (LeFevre et al., 2010).

In connection with the controversy over whether the acquisition of the cardinality principle by TD children relates more to grammar (e.g., Spelke, 2017) or to vocabulary breadth (e.g., Purpura et al., 2011; Purpura & Ganley, 2014), our results are in line with Spelke’s hypothesis, although we tested general grammatical knowledge, and not specific knowledge about quantification in language. In any case, these findings suggest that early mathematical abilities in autism may be better predicted by grammatical knowledge than by just having acquired a symbolic system, or by mastering an ample vocabulary.

Going deeper into mathematical skills, we found that relative differences of informal total scores correlated only with cognitive variables (NVIQ and reasoning), while the numbering subcategory correlated with both non-verbal intelligence and linguistic variables. Relative differences of formal total scores correlated with all the cognitive variables and relative differences in PPTV-III verbal mental age.

To our knowledge, this is the first study that examines whether the language predictors of early mathematical abilities in TD children are also good predictors of early mathematical abilities in children with autism. Our results suggest that language (grammar) and visuo-spatial cognition could predict early mathematical skills also in children with autism, independently of the severity of the condition. Therefore, assessing visuo-spatial and language abilities in children at an early age could serve as a predictive measure for future mathematical difficulties, even without an ASD diagnosis, potentially assisting those who may later receive one. Given that numerous developmental studies have demonstrated that mathematical abilities can be enhanced through instruction, implementing these interventions at an early age, before formal mathematics education in elementary schools, could be particularly beneficial.

### Limitations and Future Research

Given the variety of autistic profiles reported in other studies (e.g., Chen et al., 2019), the sample size of our study may not allow us to generalize these findings to the population of children with autism without ID. Therefore, it would be advisable to verify if these results are confirmed with a larger sample. Moreover, although standardized measures have been used to control for variables measured in typically developing children, the inclusion of a control group in this study would have enriched the findings by allowing for direct comparisons between students with and without autism. This would also provide a better understanding of whether the predictors are consistent across different groups.

On the other hand, results referring different types of mathematical abilities should be taken with caution taking into account that statistical information is not available to calculate *z*-scores. In addition, the small number of tasks expected to be solved at the considered ages for different TEMA-3 informal and formal categories does not make possible to identify which mathematical skills may be involved to a greater extent in the observed relations. For future studies, it would be advisable to discern which specific early mathematical skills are most strongly linked to grammatical knowledge, which ones to vocabulary, and which ones to non-verbal intelligence factors. Concerning grammatical knowledge, it would also be interesting to test what kind of grammatical knowledge relates to mathematical abilities, and, in particular, whether it is the grammar of quantified phrases that best predicts success, as Spelke and collaborators hold.

It is crucial to understand the impact of linguistic and cognitive factors on early mathematical skills in order to design tailored interventions that reinforce these aspects, which may hinder the early foundation of mathematical learning in children with autism. Future work should also investigate the relation between early mathematical abilities and the mathematical performance of children with autism at school, which has been shown to be generally an issue (Titeca et al., 2014). Finally, conducting longitudinal studies with individuals with autism would be commendable to validate the findings of this study and identify predictive linguistic and cognitive abilities for different profiles of mathematical performance.

## Footnotes

1 These variables are not taken to be the only possible factors underlying math skills. As Purpura et al. (2020), among others, highlight, a child’s learning environment and everyday knowledge also play crucial roles in number sense development.
